# Comparison between an exact and a heuristic neural mass model with second-order synapses

**DOI:** 10.1007/s00422-022-00952-7

**Published:** 2022-12-01

**Authors:** Pau Clusella, Elif Köksal-Ersöz, Jordi Garcia-Ojalvo, Giulio Ruffini

**Affiliations:** 1 Department of Medicine and Life Sciences, Universitat Pompeu Fabra, Barcelona Biomedical Research Park, 08003 Barcelona, Spain; 2 LTSI - UMR 1099, INSERM, Univ Rennes, Campus Beaulieu, 35000 Rennes, France; 3 Brain Modeling Department, Neuroelectrics, Av. Tibidabo, 47b, 08035 Barcelona, Spain

## Abstract

Neural mass models (NMMs) are designed to reproduce the collective dynamics of neuronal populations. A common framework for NMMs assumes heuristically that the output firing rate of a neural population can be described by a static nonlinear transfer function (NMM1). However, a recent exact mean-field theory for quadratic integrate-and-fire (QIF) neurons challenges this view by showing that the mean firing rate is not a static function of the neuronal state but follows two coupled nonlinear differential equations (NMM2). Here we analyze and compare these two descriptions in the presence of second-order synaptic dynamics. First, we derive the mathematical equivalence between the two models in the infinitely slow synapse limit, i.e., we show that NMM1 is an approximation of NMM2 in this regime. Next, we evaluate the applicability of this limit in the context of realistic physiological parameter values by analyzing the dynamics of models with inhibitory or excitatory synapses. We show that NMM1 fails to reproduce important dynamical features of the exact model, such as the self-sustained oscillations of an inhibitory interneuron QIF network. Furthermore, in the exact model but not in the limit one, stimulation of a pyramidal cell population induces resonant oscillatory activity whose peak frequency and amplitude increase with the self-coupling gain and the external excitatory input. This may play a role in the enhanced response of densely connected networks to weak uniform inputs, such as the electric fields produced by noninvasive brain stimulation.

## Introduction

Neural mass models (NMMs) provide a physiologically grounded description of the average synaptic activity and firing rate of neural populations (Wilson and Cowan 1972; Lopes da Silva et al. 1974, 1976; Jansen et al. 1993; Jansen and Rit 1995; Wendling et al. 2002). First developed in the 1970s, these models are increasingly used for both local and whole-brain modeling in, e.g., epilepsy (Wendling et al. 2002; Wendling and Chauvel 2008; Jedynak et al. 2017) or Alzheimer’s disease (Pons et al. 2010; Stefanovski et al. 2019), and for understanding and optimizing the effects of transcranial electrical stimulation (tES) (Molaee-Ardekani et al. 2010; Merlet et al. 2013; Kunze et al. 2016; Ruffini et al. 2018; Sanchez-Todo et al. 2018). However, they are only partly derived from first principles. While the post-synaptic potential dynamics are inferred from data and can be grounded on diffusion physics (Destexhe et al. 1998; Pods et al. 2013; Ermentrout and Terman 2010), the transfer function linking the mean population membrane potential with the corresponding firing rate (Freeman’s “wave-to-pulse” sigmoid function) rests on a weaker theoretical standing (Wilson and Cowan 1972; Freeman 1975; Kay 2018; Eeckman and J 1991). This results in a limited understanding on the range of applicability of the theory. For example, although models for the effects of an electric field at the single neuron are now available (Aberra et al. 2018; Galan 2021), it is unclear how they should be used at the population-level representation.

In 2015, Montbrió, Pazó, and Roxin (MPR) (Montbrió et al. 2015) derived an exact mean-field theory for networks of quadratic integrate-and-fire (QIF) neurons, thereby connecting microscale neural mechanisms with mesoscopic brain activity. Within this framework, the response of a neural population is described by a low-dimensional system representing the dynamics of the firing rate and mean membrane potential. Therefore, the MPR equations can be seen to replace the usual static transfer sigmoid function with two differential equations grounded on the biophysics of the single neurons. Since then, the theory has been applied to cover increasingly complex formulations of the single-neuron activity, including time delays (Pazó and Montbrió 2016; Devalle et al. 2018; Ratas and Pyragas 2018), dynamic synapses (Montbrió et al. 2015; Ratas and Pyragas 2016; Devalle et al. 2017; Dumont and Gutkin 2019; Coombes and Byrne 2019; Byrne et al. 2020, 2022; Avitabile et al. 2022), gap-junctions (Laing 2015; Pietras et al. 2019), stochastic fluctuations (Ratas and Pyragas 2019; Goldobin et al. 2021; Clusella and Montbrió 2022), asymmetric spikes (Montbrió and Pazó 2020), sparse connectivity (di Volo and Torcini 2018; Bi et al. 2021), and short-term plasticity (Taher et al. 2020, 2022).

In the limit of very slow synapses, the firing rate of the MPR formulation can be cast as a static function of the input currents, in the form of a population-wide *f*-*I* curve (Devalle et al. 2017). This function can be used to derive a NMM with exponentially decaying synapses, which fails to reproduce the dynamical behavior of the exact mean-field theory, highlighting the importance of the dynamical equations in the MPR model (Devalle et al. 2017). In fact, empirical evidence suggests that post-synaptic currents display rise and a decay time scales (Lopes da Silva et al. 1974; Jang et al 2010). These types of synaptic dynamics can be modelled through a second-order linear equation, which forms the basis for many NMMs (see, e.g., Lopes da Silva et al. (1974); Jansen et al. (1993); Wendling et al. (2002)). This has been also noticed by other researchers, who have recently studied exact NMMs with second-order synapses (Coombes and Byrne 2019; Byrne et al. 2020, 2022). However, a formal comparison between the MPR formalism with second-order synaptic dynamics and classical, heuristic NMMs has not yet been established.

In this paper, we analyze the NMM that results from applying the mean-field theory to a population of QIF neurons with second-order equations for the synaptic dynamics. The resulting NMM, which we refer to as NMM2 in what follows, contains two relevant time scales: one for the post-synaptic activity and one for the membrane dynamics. These two time scales naturally bridge the Freeman “wave-to-pulse” function with the nonlinear dynamics of the firing rate. In particular, following Devalle et al. (2017), we show that, in the limit of very slow synapses and external inputs, the mean membrane potential and firing rate dynamics become nearly stationary. This allows us to develop an analogous NMM with a static transfer function, which we will refer to as NMM1 for brevity. Next, we analyze the dynamics of the two models using physiological parameter values for the time constants, in order to assess the validity of the formal mapping. Bifurcation analysis of the two systems shows that the models are not equivalent, with NMM2 presenting a richer dynamical repertoire, including resonant responses to external stimulation in a population of pyramidal neurons, and self-sustained oscillatory states in inhibitory interneuron networks.

## Models

### NMM with static transfer function

Semi-empirical “lumped” NMMs where first developed in the early 1970s by Wilson and Cowan (Wilson and Cowan 1972), Freeman (Freeman 1972, 1975), and Lopes da Silva (Lopes da Silva et al. 1974). This framework is based on two key conceptual elements. The first one consists of the filtering effect of synaptic dynamics, which transforms the incoming activity (quantified by firing rate) into a mean membrane potential perturbation in the receiving population. The second element is a static transfer function that transduces the sum of the membrane perturbations from synapses and other sources into an output mean firing rate (see Grimbert and Faugeras (2006) for a nice introduction to the Jansen-Rit model). We next describe these two elements separately.

The synaptic filter is instantiated by a second-order linear equation coupling the mean firing rate of arriving signals *r* (in kHz) to the mean post-synaptic voltage perturbation *u* (in mV) (Grimbert and Faugeras 2006; Ermentrout and Terman 2010):

1 $$\begin{aligned} \tau _s^2 \ddot{u} = C\gamma r(t) -2\tau _s\dot{u} - u \end{aligned}$$

Here the parameter $\tau _s$ sets the delay time scale (ms), $\gamma$ characterizes the amplification factor in mV/kHz, and *C* is dimensionless and quantifies the average number of synapses per neuron in the receiving population. Upon inserting a single Dirac-delta-like pulse rate at time $t=0$, the solution of (1) reads $u(t)=C\gamma \tau _s^{-2}te^{-t/\tau _s}$ for a system initially at rest ($\dot{u}(0)=u(0)=0$). This model for PSPs activity is a commonly used particular case of a more general formulation that considers different rise and decay times for the post-synaptic activity (Ermentrout and Terman 2010).

The synaptic transmission equation needs to be complemented by a relationship between the level of excitation of a neural population and its firing rate, namely a transfer function, $\Phi$. Through the transfer function, each neuron population converts the sum of its input currents, *I*, to an output firing rate *r* in a nonlinear manner, i.e., $r(t) = \Phi [ I(t)]$. Wilson and Cowan, and independently Freeman, proposed a sigmoid function as a simple model to capture the response of a neural mass to inputs, based on modeling insights and empirical observations (Wilson and Cowan 1972; Freeman 1975; Eeckman and J 1991). A common form for the sigmoid function is

2 $$\begin{aligned} {{\,\textrm{Sigm}\,}}[ I] = \frac{2e_0}{1+e^{\rho (I_{0}- I)}}\,, \end{aligned}$$

where $e_0$ is the half-maximum firing rate of the neuronal population, $I_0$ is the threshold value of the input (when the firing rate is $e_0$), and $\rho$ determines the slope of the sigmoid at that threshold. Beyond this sigmoid, transfer functions can be derived from specific neural models such as the leaky integrate-and-fire or the exponential integrate-and-fire, either analytically or numerically fitting simulation data, see, e.g., Fourcaud-Trocmé et al. (2003); Brunel and Hakim (2008); Pereira and Brunel (2018); Ostojic and Brunel (2011); Carlu et al. (2020). In some studies, $\Phi$ is regarded as a function of mean membrane potential instead of the input current (Jansen and Rit 1995; Wendling et al. 2002). Nonetheless, the relation between input current and mean voltage perturbation is often assumed to be linear, see for instance Ermentrout and Terman (2010). Therefore, the difference between both formulations might be relevant only in the case where the transfer function has been experimentally or numerically derived.

The form of the total input current in Eq. (2) will depend on the specific neuronal populations being considered, and on the interactions between them. In what follows, we focus on a single population with recurrent feedback and external stimulation. Hence, the total input current is given as the contribution of three independent sources,

3 $$\begin{aligned} I(t) = \kappa u(t) + p + I_E(t) \end{aligned}$$

where $\kappa$ is the recurrent conductance, *p* is a constant baseline input current, and $I_E$ stands for the effect of an electric field. Note that some previous studies do not use an explicit self-connectivity as an argument of the transfer function (see, e.g., Grimbert and Faugeras (2006); Wendling et al. (2002); Lopez-Sola et al. (2022)). In the next section, we show that the term $\kappa u(t)$ in (3) arises naturally in recurrent networks.

Finally, we rescale the postsynaptic voltage by defining $s=u/(C\gamma )$ and use the auxiliary variable *z* to write Eq. (1) as a system of two first-order differential equations. With those choices, the final closed formulation for the neural population dynamics reads

4 $$\begin{aligned} \begin{aligned} \tau _s \dot{s}&= z \\ \tau _s \dot{z}&= \Phi [ K s(t) + p + I_E(t)] - 2z -s \; \end{aligned} \end{aligned}$$

where $K=C\gamma \kappa$. We refer to this model in what follows as NMM1.

### Quadratic integrate-and-fire neurons and NMM2

Consider a population of fully and uniformly connected QIF neurons indexed by $j=1, ..., N$. The membrane potential dynamics of a single neuron in the population, $U_j$, is described by (Latham et al. (2000); Devalle et al. (2017))

5 $$\begin{aligned} c\dot{U}_j= g_L \frac{(U_j-U_r)(U_j-U_t)}{U_t-U_r} + I_{j,\text {total}}(t)\;, \end{aligned}$$

with $U_j$ being reset to $U_\text {reset}$ when $U_j \ge U_\text {apex}$. In this equation, $U_r$ and $U_t>U_r$ represent the resting and threshold potentials of the neuron (mV), $I_{j,\text {total}}$ the input current ($\mu$A), *c* the membrane capacitance ($\mu$F), and $g_L$ is the leak conductance (mS). If unperturbed, the neuron membrane potential tends to the resting state value $U_r$. In the presence of input current, the membrane potential of the neuron $U_j$ can grow and surpass the threshold potential $U_t$, at which point the neuron emits a spike. An action potential is produced when $U_j$ reaches a certain apex value $U_\text {apex}>U_t$, at which point $U_j$ is reset to $U_\text {reset}$.

The total input current of neuron *j* is

6 $$\begin{aligned} I_{j,\text {total}}(t)=\chi _j(t) + \kappa u(t) + \tilde{I}_E(t)\;. \end{aligned}$$

The first term in this expression, $\chi _j(t)$, corresponds to a Cauchy white noise with median $\overline{\chi }$ and half-width at half-maximum $\Gamma$ (see Clusella and Montbrió (2022)). The second term, $\kappa u(t)$, represents the mean synaptic transmission from other neurons *u*(*t*), with coupling strength $\kappa$. As in NMM1, we assume that *u*(*t*) follows Eq. (1). However, in this case, the firing rate is determined self-consistently from the population dynamics as

7 $$\begin{aligned} r(t)=\frac{1}{N}\lim _{\tau _r\rightarrow 0}\sum _{j=1}^N \frac{1}{\tau _r}\sum _{k} \int _{t-\tau _r}^t \delta (t'-t_j^{(k)})\, d t' \end{aligned}$$

where $t_j^{(k)}$ is the time of the *k*th spike of neuron *j*, and the spike duration time $\tau _r$ needs to assume small finite values in numerical simulations. Finally, $\tilde{I}_E(t)$ can represent both a common external current from other neural populations, or the effect of an electric field. In the case of an electric field, the current can be approximated by $\tilde{I}_E = \tilde{P}\cdot E$, where $\tilde{P}$ is the dipole conductance term in the spherical harmonic expansion of the response of the neuron to an external, uniform electric field (Galan 2021). This is a good approximation if the neuron is in a subthreshold, linear regime and the field is weak, and can be computed using realistic neuron compartment models. We assume here for simplicity that all the QIF neurons in the population are equally oriented with respect to the electric field (this could be generalized to a statistical dipole distribution).

In order to analyze the dynamics of the model it is convenient to cast it in a reduced form. Following Devalle et al. (2017), we define the new variables

8 $$\begin{aligned} V_j=\left( U_j-\frac{U_r+U_t}{2}\right) /(U_t-U_r)\;, \,\, s=\frac{u}{C\gamma } \end{aligned}$$

and redefine the system parameters (all dimensionless except for $\tau _m$) as

9 $$\begin{aligned} \begin{aligned} \tau _m&={c/g_L}\;,\\ J&=\kappa \frac{C\gamma }{c(U_t-U_r)} \;,\\ \eta&=\frac{\zeta }{g_L(U_t-U_r)}-\frac{1}{4}\;, \\ \Delta&=\frac{\Gamma }{g_L(U_t-U_r)}\;, \\ I_E(t)&=\frac{\tilde{I}_E(t)}{g_L(U_t-U_r)}\;,\text { and} \\ \xi _j(t)&=\frac{ \chi _j(t)}{g_L(U_t-U_r)}\;. \end{aligned} \end{aligned}$$

With these transformations, the QIF model can be written as

10 $$\begin{aligned} \tau _m \dot{V}_j = V_j^2+\eta + J\tau _m s + \xi _j + I_E(t) \;. \end{aligned}$$

The synaptic dynamics are given by

11 $$\begin{aligned} \begin{aligned} \tau _s \dot{s}&= z \\ \tau _s \dot{z}&= r - 2z -s \;. \end{aligned} \end{aligned}$$

These transformations express the QIF variables and parameters with respect to reference values of time ($c/g_L$), voltage ($U_t-U_r$), and current ($g_L(U_t-U_r)$). In the new formulation, the only dimensional quantities have units of time ($\tau _m$ and $\tau _s$, in ms) or frequency (*r* and *s*, in kHz). It is important to keep in mind these changes when dealing with multiple interacting populations involving different parameters, and also when using empirical measurements to determine specific parameter values.

#### Exact mean-field equations with second order synapses (NMM2)

Starting from Eq. (10), Montbrió et al. (2015) derived an effective theory of fully connected QIF neurons in the large *N* limit. Initially, the theory was restricted to deterministic neurons with Lorentzian-distributed currents. Recently it has also been shown to apply to neurons under the influence of Cauchy white noise, a type of Lévy process that renders the problem analytically tractable (Clusella and Montbrió 2022). In any case, the macroscopic activity of a population of neurons given by Eq. (10) can be characterized by the probability of finding a neuron with membrane potential *V* at time *t*, *P*(*V*, *t*). In the limit of infinite number of neurons ($N\rightarrow \infty$), the time evolution of such probability density is given by a fractional Fokker-Planck equation (FFPE). Assuming that the reset and threshold potentials for single neurons are set to $V_\text {apex}=-V_\text {reset}=\infty$, the FFPE can be solved by considering that *P* has a Lorentzian shape in terms of a time-depending mean membrane potential *v*(*t*) and mean firing rate *r*(*t*),

12 $$\begin{aligned} P(V,t)=\frac{\tau _m r(t)}{[V-v(t)]^2+(\pi r(t) \tau _m)^2}\;, \end{aligned}$$

with

13 $$\begin{aligned} \begin{aligned} \tau _m \dot{r}&= \frac{\Delta }{\pi \tau _m} + 2rv\\ \tau _m \dot{v}&= \eta - (\pi r \tau _m)^2 + v^2 +\tau _m J s + I_E(t)\;.\\ \end{aligned} \end{aligned}$$

Together with the synaptic dynamics (11), these equations describe an exact NMM, which we refer to as NMM2.

## Slow and fast synapse dynamics limits

### Slow synapse limit and map to NMM1

Comparing the formulations of the semi-empirical model NMM1 (4) and the exact mean-field model NMM2 (13) one readily observes that the latter can be interpreted as an extension of the former. The synaptic dynamics are given by the same equations in both models, yet in NMM2 the firing rate *r* is not a static function of the input currents, but a system variable. Moreover, NMM2 includes the dynamical effect of the mean membrane potential, *v*, which in the classical framework is assumed to be directly related to the post-synaptic potential.

Devalle et al. (2017) showed that in a model with exponentially decaying (i.e., first-order) synapses, the firing rate can be expressed as a transfer function in the limit of slowly decaying synapses. Their work follows from previous results showing that, in class 1 neurons, the slow synaptic limit allows one to derive firing rate equations for the population dynamics (Ermentrout 1994). Here we revisit the same steps to show that NMM2 can be formally mapped to a NMM1 form. We perform such derivation in the absence of external inputs ($I_E(t)=0$).

Let us rescale time in Eq. (13) to units of $\tau _s$, and the rate variables to units of $1/\tau _m$ using

14 $$\begin{aligned} \tilde{t}=\frac{t}{\tau _s},\quad \tilde{s}=\tau _m s,\quad \tilde{z}=\tau _m z,\quad \tilde{r}=\tau _m r\;. \end{aligned}$$

Additionally we define $\epsilon =\tau _m/\tau _s$. Then, the NMM2 model (Eqs. (11) and (13)) reads

15 $$\begin{aligned} \begin{aligned} \epsilon \frac{d\tilde{r}}{d\tilde{t}}&= \frac{\Delta }{\pi } + 2\tilde{r}v\\ \epsilon \frac{dv}{d\tilde{t}}&= \eta + v^2-(\pi \tilde{r})^2 + J \tilde{s} \\ \frac{d\tilde{s}}{d\tilde{t}}&= \tilde{z} \\ \frac{d\tilde{z}}{d\tilde{t}}&= \tilde{r} - 2\tilde{z} -\tilde{s} \;. \end{aligned} \end{aligned}$$

Taking now $\epsilon \rightarrow 0$ ($\tau _s \rightarrow \infty$), the equations for $\tilde{r}$ and *v* become quasi-stationary in the slow time scale, i.e.,

16 $$\begin{aligned} \begin{aligned} 0&\approx \frac{\Delta }{\pi } + 2\tilde{r}v\\ 0&\approx \eta + v^2-(\pi \tilde{r})^2 +J \tilde{s}\;. \end{aligned} \end{aligned}$$

The solution of these equations is given by $\tilde{r}=\Psi _\Delta \left( \eta +J \tilde{s}\right)$, where

17 $$\begin{aligned} \Psi _\Delta (I)=\frac{1}{\pi \sqrt{2}}\sqrt{I+\sqrt{I^2 + \Delta ^2}}\;. \end{aligned}$$

This is the transfer function of the QIF model, which relates input currents to the output firing rate. Thus, in the limit $\epsilon \rightarrow 0$ system (15) formally reduces to the NMM1 formulation, Eq. (4).

In the following sections, we study to what extent this equivalence remains valid for finite ratios of $\tau _m/\tau _s$. To that end, it is convenient to recast the analogy between NMM1 and NMM2 in terms of the non-rescaled quantities, which corresponds to using

18 $$\begin{aligned} \Phi = \tau _m^{-1} \Psi _\Delta ,\; K= J\tau _m,\text { and }p=\eta , \end{aligned}$$

in Eq. (4).

**Fig. 1:**
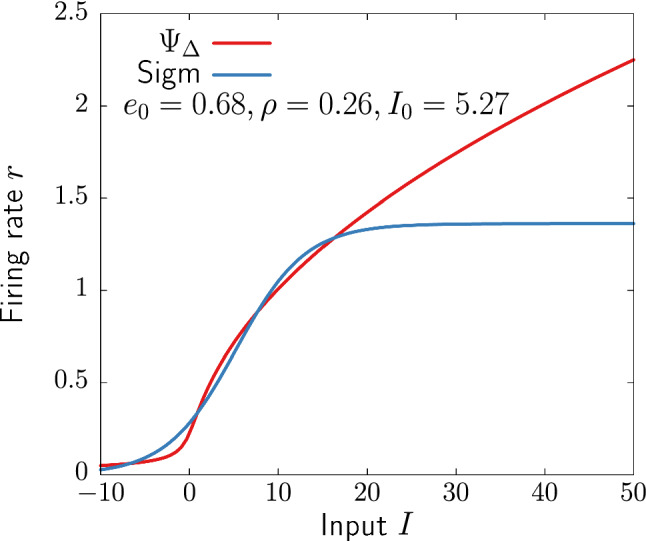
Transfer function of the QIF network (17) with $\Delta =1$ and the sigmoid (2) with parameters fitted to $\Psi _\Delta$

In Fig. 1, we fit the parameters of the sigmoid function to $\Psi _\Delta$ for $\Delta =1$. Despite the sudden sharp increase of both functions, there is an important qualitative difference: the *f*-*I* curve of the QIF model does not saturate for $I\rightarrow \infty$. Other transfer functions derived from neural models share a similar non-bounded behavior (Fourcaud-Trocmé et al. 2003; Carlu et al. 2020). This reflects the continued increase of firing activity with increase input, which has been reported in experimental studies (Rauch et al. 2003).

### Fast synapse limit

To explore the fast synapse limit, it is convenient to rescale time as $\overline{t}=t/\tau _m$ in the NMM2 equations. In this new frame, and defining $\delta :=\tau _s/\tau _m = 1/\epsilon$, the system reads

19 $$\begin{aligned} \begin{aligned} \frac{d\tilde{r}}{d\overline{t}}&= \frac{\Delta }{\pi } + 2\tilde{r}v\\ \frac{dv}{d\overline{t}}&= \eta + v^2-(\pi \tilde{r})^2 + J \tilde{s} \\ \delta \frac{d\tilde{s}}{d\overline{t}}&= \tilde{z} \\ \delta \frac{d\tilde{z}}{d\overline{t}}&= \tilde{r} - 2\tilde{z} -\tilde{s} \;. \end{aligned} \end{aligned}$$

where $\tilde{r}$, $\tilde{s}$ and $\tilde{z}$ are the rescaled variables defined in (14). With the algebraic conditions in the fast synapse limit, $\delta \rightarrow 0$ ($\tau _s \rightarrow 0$), Eq. (19) is reduced to

20 $$\begin{aligned} \begin{aligned} \frac{d\tilde{r}}{d\overline{t}}&= \frac{\Delta }{\pi } + 2\tilde{r}v\\ \frac{dv}{d\overline{t}}&= \eta + v^2-(\pi \tilde{r})^2 + J \tilde{r} \;, \end{aligned} \end{aligned}$$

where we have used that $\tilde{s}=\tilde{r}$ as given by the synaptic equations. This is the model with instantaneous synapses analyzed by Montbrió et al. (2015), who showed that the $\eta$–*J* phase diagram has three qualitatively distinct regions in the presence of a constant input: a single stable node corresponding to a low-activity state, a single stable focus (spiral) generally corresponding to a high-activity state, and a region of bistability between a low activity steady state and a regime of asynchronous persistent firing.

## System dynamics

In the previous section, we have shown that NMM2 can be mapped to NMM1 in the limit of slow synapses ($\tau _m/\tau _s\rightarrow 0$), using the scaling relations (18). However, physiological values for the time constants might not be consistent with this limit. Table 1 shows reference values for $\tau _m$ and $\tau _s$ corresponding to different neuron types and their corresponding neurotransmitters obtained from experimental studies. Notice that, in practice, such values also depend on the electrical and morphological properties of the neurons, and pre- and post-neuron types. Such level of detail requires the use of conductance-based compartmental models, a further step in mathematical complexity that is out of the scope of this paper. Therefore, we take the values in Table 1 as coarse-grained quantities that properly reflect the time scales in point neuron models such as the QIF (10) (for a more detailed discussion, see Sect. 5). In order to study to what extent these non-vanishing values of $\tau _m/\tau _s$ break down the equivalence between the two models, in this section we analyze and compare the dynamics of a single neural population with recurrent connectivity described by both NMM1 (Eq. (4) with Eq. (18)) and NMM2 (Eqs. (11) and (13)).

**Table 1:** Values for the membrane time constants $\tau _m$ and postsynaptic currents $\tau _s$, for pyramidal neurons (Pyr), parvalbumin-positive (PV+), and neurogliaform cells (NGFC) (Neske et al. 2015; Zaitsev et al. 2012; Povysheva et al. 2007; Avermann et al. 2012; Oláh et al. 2007; Seay et al. 2020; Karnani et al. 2016; Bacci et al. 2003; Deleuze et al. 2019). Notice that, in general, the synaptic time-constant should depend on the neurotransmitter, and the pre- and post-synaptic cells. Since we only consider self-coupled populations, we do not specify time constants for transmission across populations of different types

Neuron type	Neurotransmitter	$\tau _m$ (ms)	$\tau _s$ (ms)	$\tau _m/\tau _s$
Pyr	Glutamate	15	10	1.5
PV+	GABA	7.5	2	3.75
NGFC	GABA	11	20	0.55

The first step is to identify the steady states of the system. Since we derived the transfer function (17) by assuming the *r* and *v* variables of NMM2 to be nearly stationary, the fixed points of both models coincide and are given by:

21 $$\begin{aligned} \begin{aligned} \tau _m r_0&=\Psi _\Delta (\eta +\tau _m Jr_0),\\ v_0&=-\frac{\Delta }{2\tau _m \pi r_0},\\ s_0&= r_0,\;\\ z_0&=0. \end{aligned} \end{aligned}$$

Moreover, $r_0$ and $v_0$ are the equilibrium points of the two-dimensional system analyzed by Montbrió et al. (2015). Notice that the only relevant parameters for the determination of the fixed points are *J*, $\eta$, and $\Delta$. The time constant $\tau _m$ only acts as a multiplicative factor of $r_0$ (and $s_0$), and $\tau _s$ does not enter into the expressions of the steady states.

Even though the steady states of the three models (NMM1, NMM2, and the original system of Montbrió et al. (2015)) are the same, their stability properties might be different, as we now attempt to elucidate. The eigenvalues controlling the stability of the fixed points in NMM1 are:

22 $$\begin{aligned} \lambda _\pm =\tau _s^{-1}\left( -1\pm \sqrt{J\Psi _\Delta '[\eta + J\tau _m s_0 ]}\right) \end{aligned}$$

A similar closed expression for NMM2 is complicated to obtain and, in any case, there are no explicit expressions for the steady states. Thus, we use in what follows the numerical continuation software AUTO-07p (Doedel et al. 2007) to obtain the corresponding bifurcation diagrams. We analyze separately the dynamics of excitatory ($J>0$) and inhibitory ($J<0$) neuron populations in the two NMM models.

**Fig. 2:**
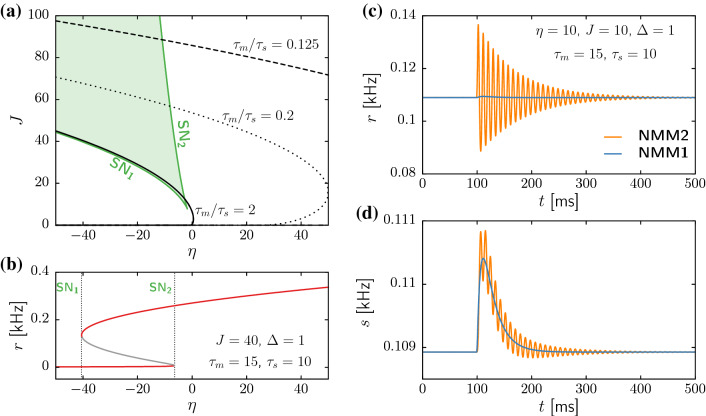
Dynamics of a population of pyramidal neurons described by NMM1 and NMM2. **a** Saddle–node bifurcations SN$_1$ and SN$_2$ (green curves) limiting the region of bistability (light-green region), and node-focus boundary for three different values of $\tau _m/\tau _s$ in NMM2 (solid, dotted and dashed black lines). **b** Steady-state value of the firing rate as a function $\eta$, for fixed $J=40$, $\Delta =1$, $\tau _m=15$ ms, and $\tau _s=10$ ms. The stable steady-state branches are colored in red, and the unstable steady-state branch in grey. Dashed vertical lines indicate the SN1 and SN2 bifurcation points (cf. panel a). **c**, **d** Time evolution of the firing rate *r* **c** and synaptic variable *s* **d** for NMM1 (blue) and NMM2 (orange), for $\eta =10$, $J=10$, $\Delta =1$, $\tau _m=15$ms, and $\tau _s=10$ ms. Initial conditions are at steady state, and a 1-ms-long pulse of $I_E(t)=10$ is applied at $t=100$ ms

### Pyramidal neurons

We start by analyzing the dynamics of NMM1 in the case of excitatory coupling ($J>0$), by fixing $\Delta =1$ and varying $\eta$ and *J*. Following Table 1, we set $\tau _m=15$ ms and $\tau _s=10$ ms. Since the NMM1 eigenvalues (22) are real for $J>0$, the fixed points do not display resonant behavior, i.e., they are either stable or unstable nodes. For positive baseline input $\eta$, only a single fixed point exists irrespective of the value of the coupling *J*. In contrast, a large region of bistability bounded by two saddle–node (SN) bifurcations emerges for negative $\eta$. The green curves in Fig. 2a show the two SN bifurcations, which merge in a cusp close to the origin of parameter space. Within the region bounded by the curves (green shaded region), a low-activity and a high-activity state coexist, separated by a third unstable fixed point. Figure 2b displays, for instance, the stationary firing rate as a function of $\eta$ for $J=40$. The values of the time constants $\tau _m$ and $\tau _s$ do not affect the bistability region. However, the noise amplitude $\Delta$ does have an effect: as shown in Appendix A, NMM1 admits a parameter reduction that expresses all parameters and variables as functions of $\Delta$. Accordingly, the effects of modifying $\Delta$ on the stability of the fixed points are analogous to rescaling $\eta \rightarrow \eta /\Delta$ and $J\rightarrow J/\sqrt{\Delta }$ (see also Montbrió et al. (2015)). Therefore, the bistable region shrinks in the $(\eta ,J)$ parameter space as the noise amplitude increases.

Since the fixed points of NMM1 and NMM2 coincide, these two branches of SN bifurcations also exist in NMM2. Moreover, no other bifurcations arise; thus, the diagrams depicted in Fig. 2a,b also hold for the exact model. However, there is an important difference regarding the relaxation dynamics towards the fixed points: While in NMM1 the steady states are always nodes, in NMM2 trajectories near the high-activity state might display transient oscillatory behavior. Figure 2c,d display, for instance, time series obtained from simulations of NMM1 (blue) and NMM2 (orange) starting at the fixed point, and receiving a small pulse applied at $t=100$ ms. Not only NMM2 displays an oscillatory response, but also the effect of the perturbation in the firing rate is much larger in NMM2 than in NMM1.

Such resonant behavior of NMM2 corresponds to the two dominant eigenvalues of the high-activity fixed point (those with largest real part) being complex conjugates of each other. The black curves in Fig. 2a show the boundary line at which those two eigenvalues change from real (below the curves) to complex (above the curves). For physiological values of $\tau _m$ and $\tau _s$ (continuous black line), this node-focus line remains very similar to that of the model with instantaneous synapses studied in Montbrió et al. (2015). Reducing the ratio $\tau _m/\tau _s$ changes this situation. As shown by the dotted and dashed curves in Fig. 2a, as we approach the slow synaptic limit ($\tau _m/\tau _s\rightarrow 0$) the resonant region (where the dominant eigenvalues are complex) requires increasingly larger values of $\eta$ and $\Delta$, vanishing for small enough ratio $\tau _m/\tau _s$. Hence, as expected from the time scale analysis of Sect. 3, the dynamics of NMM2 can be faithfully reproduced by NMM1 in this limit. However, the equivalence cannot be extrapolated to physiological parameter values.

### Interneurons

Here we consider a population of GABAergic interneurons with self-recurrent inhibitory coupling ($J<0$). In particular, we focus on parvalbumin-positive (PV+) fast spiking neurons, which play a major role in the generation of fast collective brain oscillations (Bartos et al. 2002, 2007; Cardin et al. 2009; Tiesinga and Sejnowski 2009). We thus set $\tau _m=7.5$ and $\tau _s=2$ ms, following Table 1.

In this case, the NMM1 dynamics are rather simple: there is a single fixed point that remains stable, with a pair of complex conjugate eigenvalues (see Eq (22)). Therefore, the transient dynamics do display resonant behavior upon external perturbation. Nonetheless, no self-sustained oscillations emerge.

**Fig. 3:**
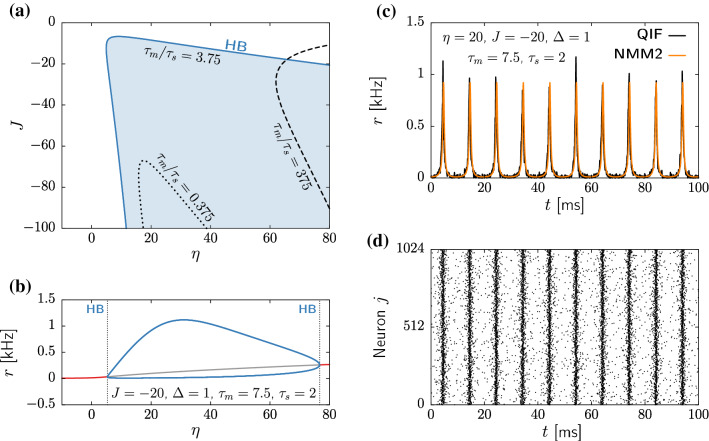
Dynamics of a population of parvalbumin-positive interneurons described by NMM2. **a** Supercritical Hopf bifurcation signaling the onset of oscillatory activity for $\tau _m=7.5$ ms, $\tau _s=2~ms$ (blue curve), $\tau _m=7.5$ ms, $\tau _s=20$ ms (dotted black curve), and $\tau _m=7.5$ ms, $\tau _s=0.02$ ms (dashed black curve). The blue-shaded region indicates stable limit-cycle behavior for $\tau _m=7.5$ ms, $\tau _s=2$ ms. **b** Steady-state values of the firing rate as a function of the input $\eta$, for fixed $J=-20$, $\Delta =1$, $\tau _m=7.5$ ms, and $\tau _s=2$ ms. The red line represents the stable steady state, the grey line the unstable steady state, and the blue lines the maxima and minima of the stable limit-cycle. Dashed vertical lines indicate the location of supercritical Hopf bifurcations (cf panel a). **c** Time evolution of the firing rate *r* for an inhibitory population at the oscillatory state ($\eta =20$, $J=-20$, $\Delta =1$, $\tau _m=7.5$ ms, and $\tau _s=2$ ms) obtained from integrating the a network with $N=1024$ QIF neurons (10) (black) and from the NMM2 (13) (orange). **d** Raster plot of the spiking times in the simulation of the QIF network corresponding to panel (**c**). Simulations of QIF network were performed with $V_\text {apex}=-V_\text {reset}=100$ using Euler–Maruyama integration with $dt=10^{-3}$ ms. The firing rate *r* is computed using Eq. (7) with $\tau _r=10^{-2}$ ms

In the NMM2, however, the unique fixed point might lose stability for $\eta >0$ through a supercritical Hopf bifurcation (HB+, see blue curve in Fig. 3a). This transition gives rise to a large region of fast oscillatory activity, corresponding to the so-called interneuron-gamma (ING) oscillations (Whittington et al. 1995; Traub et al. 1998; Whittington et al. 2000; Bartos et al. 2007; Buzsáki and Wang 2012). An example of this regime is shown in Fig. 3b,c, using both NMM2 and microscopic simulations of a QIF network as defined by Eq. (10) .

According to the ING mechanism, oscillations emerge due to a phase lag between two opposite influences: the noisy excitatory driving (controlled by $\eta$ and $\Delta$) and the strong inhibitory feedback from the recurrent connections (controlled by *J*). In NMM2, the dephasing between these two forces stems from the implicit delay caused by the synaptic dynamics. Hence, the ratio between membrane and synaptic characteristic times, $\tau _m$ and $\tau _s$, has a fundamental role in the generation of ING oscillations. The blue region depicted in Fig. 3a corresponds to time scales of PV+ neurons, $\tau _m=7.5$ and $\tau _s=2$. In this case, the oscillation frequency is in the gamma range (40-200Hz). However, by decreasing the parameter ratio $\tau _m/\tau _s$ the Hopf bifurcation becomes elusive, as the oscillatory region shrinks, and oscillations require stronger inhibitory feedback (see black dotted curve in Fig. 3a). Similarly, by increasing $\tau _m/\tau _s$ the ING activity also fades, as larger inputs $\eta$ are required to produce oscillatory activity (see black dashed curve in Fig. 3a). As showed in the previous section, the two limits of $\tau _m/\tau _s$ coincide with NMM1 and the model analyzed in Montbrió et al. (2015). The results presented above show that the membrane and synaptic dynamics are required to have comparable time scales, in order to generate oscillatory activity in NMM2.

### Network-enhanced resonance in excitatory populations

The bifurcation analysis of Sect. 4.1 reveals that a single population of excitatory neurons does not display self-sustained oscillations in neither NMM1 nor NMM2. This is expected, as excitation alone is known to be usually insufficient for the emergence of collective rhythms (Van Vreeswijk et al. 1994). However, in NMM2, the high-active steady state corresponds to a stable focus in a large region of the parameter space. In this section, we exploit this resonant behavior, inspired by the oscillatory response of a population of pyramidal neurons subject to tACS stimulation. We thus consider the NMM2 model with $\tau _m=15$ ms and $\tau _s=10$ ms injected with a current

23 $$\begin{aligned} I_E(t)=A\sin (\omega t)\;. \end{aligned}$$

We expect to induce oscillatory activity if $\omega$ is close to the resonant frequency of the system, given by $\nu :={{\,\textrm{Im}\,}}[\lambda ]$, where $\lambda$ is the fixed point eigenvalue with largest real part.

**Fig. 4:**
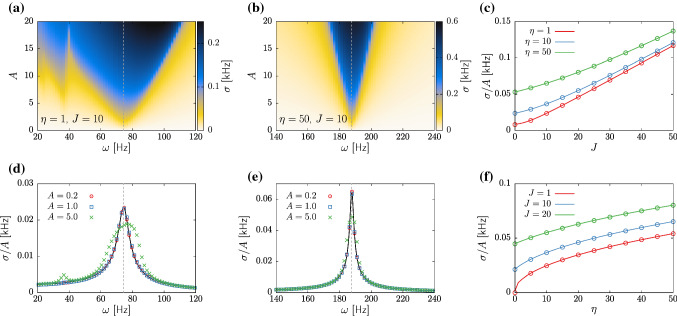
Effects of tACs stimulation, Eq. (23), in a population pyramidal neurons given by NMM2 (13). **a**, **b** Heatmaps of the standard deviation of *r* displaying Arnold tongues for $\eta =1$ (panel (**a**)) and $\eta =50$ (panel (**b**)). The rest of system parameters are $J=10$, $\Delta =1$, $\tau _m=15$ ms, and $\tau _s=10$ ms. **c** Normalized amplitude $\sigma /A$ obtained by stimulating the population at its resonant frequency $\nu$, for increasing values of the coupling strength. Continuous lines correspond to analytical results (Eq. (24)), and circles correspond to numerical simulations. **d**, **e** Normalized amplification $\sigma /A$ corresponding to the same parameters of panels (**a**) and (**b**), respectively. Symbols correspond to the numerical simulations reported in (**a**) and (**b**). The black continuous lines correspond to Eq. (24). **f** Normalized amplitude $\sigma /A$ at the resonant frequency $\nu$ upon increasing the external input $\eta$. Lines correspond to Eq. (24) and symbols to numerical simulations. In all panels, periodic stimulation has been simulated for 2 seconds after letting the system relax to the fixed point for 1 second. The reported values for the standard deviation $\sigma$ correspond only to the last 1 s of stimulation, in order to avoid capturing transient effects

Figure 4a,b displays heatmaps of the standard deviation of the firing rate, $\sigma$, obtained by stimulating the stable focus of NMM2 at different frequencies $\omega$ and amplitudes *A*. For weak baseline input $\eta$ (Fig. 4a), the amplitude of the system displays a large tongue-shaped region, with a few additional narrow tongues at smaller frequencies.

The main tongue is centered at the resonant frequency $\omega \simeq \nu$ (see grey vertical dashed line) and corresponds to entrainment at the driving rhythm, whereas secondary tongues correspond to entrainment at higher harmonics. Increasing the external input $\eta$ (Fig. 4b) causes the system to resonate at larger frequencies and shrinks the region of amplification of the applied stimulus. Despite the similitude with the usual Arnold tongues that characterize driven oscillatory systems, we recall that here we are inducing oscillatory activity in an otherwise stationary system. Hence, even if small in amplitude, there is always an oscillatory response at some harmonic of the driving frequency.

Electric stimulation protocols usually achieve large effects even when the amplitudes of the oscillatory input signal are small. We thus investigate the effect of weak stimuli through a perturbative analysis for $0<A\ll 1$. Upon expanding the NMM2 equations close to the fixed point and solving the resulting linear system, we obtain the amplitude response as a function of the driving frequency:

24 $$\begin{aligned} \begin{aligned} \mathcal {A}&(\omega ;\lambda ,\beta ) =\\&\Bigl \{ \left[ (\omega ^2+\mu ^2-\nu ^2)(\nu b_\text {i}-\mu b_\text {r}) - 2\mu \nu (\nu b_\text {r}+\mu b_\text {i}) \right] ^{2} \\&+ \omega ^2\left[ 2 b_\text {i} \mu \nu + b_\text {r}(\omega ^2+\mu ^2-\nu ^2) \right] ^{2}\Bigr \}^{1/2}\\&\times \left[ (\omega ^2+\mu ^2-\nu ^2)^2+4\mu ^2\nu ^2\right] ^{-1} \end{aligned} \end{aligned}$$

where $\lambda =\mu +i \nu$ is the fixed point eigenvalue with largest real part, and $b=b_r+ib_i$ is the associated amplitude component (see Appendix B): for the mathematical details). These two complex quantities can be obtained by numerically computing the eigenvalues and eigenvectors of the system Jacobian. The black curves in Fig. 4d and e illustrate the validity of the analytical expression when compared with numerical results (colored symbols). Overall, the perturbative analysis provides a good approximation for $A<1$, showing that, at this stage, $\omega =\nu$ provides the maximal amplification.

Finally, we use these results to investigate the effect of the system parameters *J* and $\eta$ to the amplitude response of the neural mass. Figure 4b, c shows numerical (open circles) and analytical (lines) results obtained using the optimal stimulation protocol $\omega =\nu$ with $A=0.1$ for different values of *J* and $\eta$. Overall, the oscillation amplitude of the system shows a supralinear increase with *J* and a sublinear increase with $\eta$. These results illustrate the importance of self-connectivity in tACs stimulation and can potentially explain the effectiveness of these protocols in spite of the weakness of the applied electric field. Since we only considered driving of an excitatory population, the associated resonant frequencies can be quite large (up to 400Hz for $\eta =50$ and $J=50$), which calls for future investigations to analyze the combined effect of tACs in networks with excitation-inhibition balance.

## Conclusions

For decades, NMMs have been built up on the basis of a simple framework that combines the linear dynamics of synaptic activation with a nonlinear static transfer function linking neural activity (firing rate) to excitability (Wilson and Cowan 1972; Freeman 1975; Lopes da Silva et al. 1974). This view has been sustained by empirical observations and heuristic assumptions underlying neural activity. Models based on this framework have been used to explain the mechanisms behind neural oscillations (Lopes da Silva et al. 1974; Freeman 1987; Jansen and Rit 1995; Wendling et al. 2002), and, more recently, to create large-scale brain models to address the treatment of neuropathologies by means of electrical stimulation (Kunze et al. 2016; Sanchez-Todo et al. 2018; Forrester et al. 2020).

Further theoretical efforts have provided more sophisticated tools to model the dynamics of neural populations, by deriving transfer functions from specific single-cell models (Gerstner 1995; Brunel and Hakim 2008; Ostojic and Brunel 2011; Carlu et al. 2020), add adaptation mechanisms (Augustin et al. 2017), or finite size effects (Benayoun et al. 2010; Buice et al. 2010). In this context, exact NMMs (also known as next-generation NMMs) pave a new road to directly relate single neuron dynamics with mesoscopic activity (Montbrió et al. 2015). Understanding how this novel framework relates to previous semi-empirical models should allow us to validate the range of applicability of classical NMMs.

Here we have studied a neural mass with second-order synapses, similar to the one studied in recent works (Coombes and Byrne 2019; Byrne et al. 2020, 2022). The model naturally links the dynamical firing rate dynamics derived by Montbrió et al. (2015) with the typical linear filtering representing synaptic transmission that is used in heuristic NMMs. Following Ermentrout (1994) and Devalle et al. (2017), we show that, in the slow-synapse limit and in the absence of time-varying inputs, the exact model can be formally mapped to a simpler formalism with a static transfer function. However, we find that the range of validity of this relationship is beyond the physiological values of the model parameters. An analysis of the dynamics using realistic values of the time constants illustrates the fact that fundamental properties, such as the resonant behavior of excitatory populations and the interneuron-gamma oscillatory dynamics of PV+ neurons, cannot be captured by a traditional formulation of the model.

In the context of heuristic NMMs, some works proposed to include additional adaptation variables to further fill the gap between mesoscale and single-cell models (Camera et al. 2004; Augustin et al. 2017). In spite of increased similarity with the underlying neuronal networks dynamics, the resulting equations remain steady-state approximations, and their accuracy are model- and parameter-dependent. Interestingly, analogous spike-adaptation mechanisms improve accuracy of integrate-and-fire models in single-cell studies (Rauch et al. 2003; Mensi et al. 2012); thus, similar mechanisms have also been considered in the context of NMM2 (Gast et al. 2020). Nonetheless, a systematic comparison of the effect of adaptation in the two frameworks is missing.

Despite the exact mean-field theory leading to NMM2 is a major step forward on the development of realistic mesoscale models for neural activity, the QIF neuron is a simplified model with some limitations. For instance, here we have employed non-refractory neurons, for which increasing input currents always lead to an increase of the firing rate. Future studies should address the role of a refractive period on the emerging rhythms and stimulation effects of exact NMMs. This could lead to a more realistic saturating shape of the QIF transfer function (Fig. 1). Additionally, further considerations may need to be taken into account in order to translate experimental observations to the model. In particular, the synapse time constants reported in Table 1 should reflect the delay and filtering associated with current transmission from input site to soma. This is not trivial to measure experimentally, and it can change considerably depending on synapse location, morphology, the number of simultaneously activated spine synapses (Eyal et al. 2018), and electrical properties (Koch and Segev 2003), which are not accounted by the QIF neuron, but can be estimated using realistic compartment models (Agmon-Snir and Segev 1993). Besides, the QIF model is an approximation of type-I excitable neurons, with type-II having a completely different firing pattern and *f*-*I* curve.

An important application of the exact mean-field theory is in the context of transcranial electrical stimulation. Several decades of research suggest that weak electric fields influence neural processing (Ruffini et al. 2020). In tES, the electric field generated on the cortex is of the order of 1 V/m, which is known to produce a sub-mV membrane perturbation (Bikson et al. 2004; Ruffini et al. 2013; Aberra et al. 2018). Yet, the applied field is mesoscopic in nature and is applied during long periods, with a spatial scale of several centimeters and temporal scales of thousands of seconds. Hence, a long-standing question in the field is how networks of neurons process spatially uniform weak inputs that barely affect a single neuron, but produce measurable effects in population recordings. By means of the exact mean-field model, we have shown that the sensitivity of the single population to such a weak alternating electric field can be modulated by the intrinsic self-connectivity and the external tonic input of the neural population in a population of excitatory neurons. Importantly, such resonant behavior cannot be captured by heuristic NMMs with static transfer functions.

For the physiologically inspired parameter values chosen in this study, the amplification effects on excitatory neurons appear to be weaker than those observed experimentally. We may conjecture that certain neuronal populations may be in states near criticality, i.e., close to the bifurcation points in the NMM2 model (Chialvo 2004; Carhart-Harris 2018; Vázquez-Rodríguez et al 2017; Zimmern 2020; Kōksal Ersōz and Wendling 2021; Ruffini and Lopez-Sola 2022). This would apply, for example, to inhibitory populations, which display a Hopf bifurcation where a state near the critical point will display arbitrarily large amplified sensitivity to weak but uniform perturbations applied over long time scales. Since electric fields are expected to couple more strongly to excitatory cells, this case should be studied in the context of a multi-population NMM2, with excitatory cells relaying the electric field perturbation. Exact NMMs provide an appropriate tool to investigate this behavior, as well as the effects of non-homogeneous electrical fields—which we leave to future studies.

## Appendix A Parameter reduction in NMM1 and NMM2

A common way to simplify the analysis of dynamical systems such as NMM1 (Eq. 4) and NMM2 (Eqs. 11,13) is through parameter reduction. While this can be achieved in different ways, here we choose, following Montbrió et al. (2015), to rescale the system parameters as follows:

A1 $$\begin{aligned} \tilde{\eta }=\frac{\eta }{\Delta },\quad \tilde{J}=\frac{J}{\sqrt{\Delta }},\quad \beta =\frac{\tau _s\sqrt{\Delta }}{\tau _m} \end{aligned}$$

We then define the new variables

A2 $$\begin{aligned} \begin{aligned} \tilde{t}=\frac{\sqrt{\Delta }}{\tau _m}t,&\quad \tilde{r}=\frac{\tau _m}{\sqrt{\Delta }}r, \quad \tilde{v}=\frac{v}{\sqrt{\Delta }},\\ \quad \tilde{s}=\frac{ \tau _m }{\sqrt{\Delta }}s,&\qquad \tilde{z}=\frac{ \tau _m }{\Delta }z\;. \end{aligned} \end{aligned}$$

With these definitions, and together with the equivalence relation (18), NMM1 takes the form:

A3 $$\begin{aligned} \begin{aligned} \beta \frac{d\tilde{s}}{d\tilde{t}}&= \tilde{z} \\ \beta \frac{d\tilde{z}}{d\tilde{t}}&= \Psi _1[\tilde{\eta }+ \tilde{J} \tilde{s}] - 2\tilde{z} -\tilde{s}\;. \end{aligned} \end{aligned}$$

Similarly, the NMM2 model (Eqs. 11,13) becomes

A4 $$\begin{aligned} \begin{aligned} \frac{d\tilde{r}}{d\tilde{t}}&= \frac{1}{\pi } + 2\tilde{r}\tilde{v}\\ \frac{d\tilde{v}}{d\tilde{t}}&= \tilde{\eta }- (\pi \tilde{r})^2 + \tilde{v}^2 +\tilde{J}\tilde{s}\\ \beta \frac{d\tilde{s}}{d\tilde{t}}&= \tilde{z} \\ \beta \frac{d\tilde{z}}{d\tilde{t}}&= \tilde{r} - 2\tilde{z} - \tilde{s} \;. \end{aligned} \end{aligned}$$

These reduced systems reveal that the dynamics of both models are controlled only by the three effective parameters $\tilde{\eta }$, $\tilde{J}$, and $\beta$. In particular, the effects of changing $\Delta$ in the attractors of the system can be achieved by appropriately modifying the other parameters. Also, this reduction makes explicit that the bifurcations of the system do not depend on specific values of $\tau _m$ and $\tau _s$, but only on their ratio. Notice, however, that in (A4) all parameters and variables, including time, become adimensional. This contrasts with the formulation used throughout the paper (Eqs. (13)), where time has units of milliseconds.

## Appendix B Analysis of a weakly periodically forced system

Here we present the results on weakly periodically perturbed systems used to investigate the response of NMM2 to periodic stimulation in Sect. 4.3. Although we have a specific system in mind, we consider a general setup for simplicity. Let $\varvec{x}(t)\in \mathbb {R}^n$ be an *n*-dimensional state vector, with time evolution given by the autonomous nonlinear system

B5 $$\begin{aligned} \dot{\varvec{x}} =\varvec{F}(\varvec{x})\;. \end{aligned}$$

In NMM2, $\varvec{F}$ follows Eqs. (11,13), and the state vector reads $\varvec{x}=(r,v,s,z)^T$. Let $\varvec{x}^{(0)}$ be a stable fixed point of the system and $\varvec{J}=\varvec{J}(\varvec{x}^{(0)})$ the corresponding Jacobian. We consider a periodic forcing acting on Eq. (B5),

$$\begin{aligned} \dot{\varvec{x}} =\varvec{F}(\varvec{x})+\epsilon \varvec{a}\sin (\omega t) \end{aligned}$$

where $\epsilon$ is a weak coupling $0< \epsilon \ll 1$ and $\varvec{a}\in \mathbb {R}^n$ is a normalized vector for the distribution of the forcing across the system variables. For instance, in the case considered in the paper $\varvec{a}=(0,1,0,0)^T$, since the periodic driving acts only on the mean membrane potential.

Let $\varvec{x}=\varvec{x}^{(0)}+\epsilon \varvec{\delta x}$. Since $\epsilon \ll 1$ we can linearize close to the fixed point, $\varvec{x}^{(0)}$, to obtain

$$\begin{aligned} \dot{\varvec{\delta x}}= \varvec{J}\varvec{\delta x} + \varvec{a}\sin (\omega t)\;. \end{aligned}$$

Let $\varvec{S}$ be the matrix of eigenvectors of $\varvec{J}$, and $\varvec{\Lambda }$ the diagonal matrix of eigenvalues, so that $\varvec{S}^{-1}\varvec{J}\varvec{S}=\varvec{\Lambda }$. The coordinates of the perturbation vector in the basis defined by the Jacobian eigenvalues read $\varvec{\alpha }:=\varvec{S}^{-1}\varvec{\delta x}$. Therefore,

B6 $$\begin{aligned} \begin{aligned} \dot{\varvec{\alpha }}&=\varvec{S}^{-1}\dot{\varvec{\delta x}}\\&=\varvec{S}^{-1} \varvec{J}\varvec{S}\varvec{S}^{-1}\varvec{\delta x} + \varvec{S}^{-1}\varvec{a}\sin (\omega t)\\&= \varvec{\Lambda } \varvec{\alpha } + \varvec{b}\sin (\omega t) \end{aligned} \end{aligned}$$

where $\varvec{b}:=\varvec{S}^{-1}\varvec{a}$, i.e., the coordinates of $\varvec{a}$ in the basis defined by $\varvec{S}$.

Since $\varvec{\Lambda }$ is a diagonal matrix, Eq. (B6) can be written in scalar form for each $\alpha _j$ in complex space as

$$\begin{aligned} \dot{\alpha }_j = \lambda _j \alpha _j + b_j \sin (\omega t)\;, \quad \text {for}\quad j=1,\dots ,n\;. \end{aligned}$$

In what follows we drop the subindices *j* for simplicity. The solution of each of the linear systems for $\alpha$ read

$$\begin{aligned} \alpha (t)= -b \frac{\omega \sin (\omega t)+\lambda \cos (\omega t)}{\lambda ^2 + \omega ^2} + k e^{\lambda t}\;. \end{aligned}$$

with $k \in \mathbb R$ a free constant. Since the fixed point is stable, the last term vanishes in the long term. Let $\lambda =\mu +i\nu$. The behavior of $\alpha$ greatly changes depending on whether $\nu$ is zero or not, i.e., whether the fixed point is a stable node or a stable focus. Let us start for the simple case, $\nu =0$. Then, at $t\rightarrow \infty$,

$$\begin{aligned} \alpha (t)=b\frac{\sin (\omega t + \phi )}{\sqrt{\mu ^2+\omega ^2}} \end{aligned}$$

where $\phi =\arctan (\mu /\omega )$. Therefore, these types of components always oscillate, but the amplitude of the oscillations decays as $1/\sqrt{\mu ^2 + \omega ^2}$. Hence, if the forcing frequency is too fast, or the stability too strong, then the induced oscillatory component becomes negligible.

Let’s turn now to the more interesting case of $\nu \ne 0$. Since we are considering a real system, there is always a pair of complex eigenvalues such that $\lambda _\pm =\mu \pm i\nu$ associated with complex conjugate eigenvectors $\alpha _\pm$ (conjugate root theorem for polynomials with real coefficients). Therefore, the dynamics of the real system is given by the real part of $\alpha _\pm$. We find that (for $t\rightarrow \infty$),

$$\begin{aligned} \frac{\alpha (t)+\alpha ^*(t)}{2} =\epsilon \mathcal {A}(\omega ;\mu ,\nu )\sin (\omega t+\phi ) \end{aligned}$$

where

B7 $$\begin{aligned} \mathcal {A}(\omega ;\mu ,\nu )=\frac{\sqrt{P^2+Q^2}}{D} \end{aligned}$$

and $$\phi =\arctan (Q/P)$$, with

$$\begin{aligned} \begin{aligned} P&:=(\omega ^2+\mu ^2-\nu ^2)(\nu b_\text {i}-\mu b_\text {r}) - 2\mu \nu (\nu b_\text {r}+\mu b_\text {i})\;, \\ Q&:= \omega [- 2 b_\text {i} \mu \nu -b_\text {r}(\omega ^2+\mu ^2-\nu ^2)]\;,\\ D&:=(\omega ^2+\mu ^2-\nu ^2)^2+4\mu ^2\nu ^2\;, \end{aligned} \end{aligned}$$

which corresponds to Eq. (24) in the main text.
